# “Anterior convergent” chest probing in rapid ultrasound transducer positioning versus formal chest ultrasonography to detect pneumothorax during the primary survey of hospital trauma patients: a diagnostic accuracy study

**DOI:** 10.1186/s13032-015-0030-5

**Published:** 2015-12-21

**Authors:** Behrad Ziapour, Houman Seyedjavady Haji

**Affiliations:** Emergency Department of Imam Khomeyni Hospital, Ahvaz Jundishapur University of Medical Sciences, Ahwaz, Iran; Internal Medicine, St. Vincent Hospital, Worcester, MA USA

**Keywords:** Ultrasonography, Pneumothorax, Diagnosis, Sensitivity and specificity, Transducer positioning, Primary survey

## Abstract

**Background:**

Occult pneumothorax represents a diagnostic pitfall during the primary survey of trauma patients, particularly if these patients require early positive pressure ventilation. This study investigated the accuracy of our proposed rapid model of ultrasound transducer positioning during the primary survey of trauma patients after their arrival at the hospital.

**Methods:**

This diagnostic trial was conducted over 12 months and was based on the results of 84 ultrasound (US) exams performed on patients with severe multiple trauma. Our index test (US) was used to detect pneumothorax in four pre-defined locations on the anterior of each hemi-thorax using the “Anterior Convergent” approach, and its performance was limited to the primary survey. Consecutively, patients underwent chest-computed tomography (CT) with or without chest radiography. The diagnostic findings of both chest radiography and chest ultrasounds were compared to the gold-standard test (CT).

**Results:**

The diagnostic sensitivity was 78 % for US and 36.4 % for chest radiography (*p* < 0.001); the specificity was 92 % for US and 98 % for chest radiography (not significant); the positive predictive values were 74 % for US and 80 % for chest radiography (not significant); the negative predictive values were 94 % for US and 87 % for chest radiography (not significant); the positive likelihood ratio was 10 for US and 18 for chest radiography (*p* = 0.007); and the negative likelihood ratio was 0.25 for US and 0.65 for chest radiography (*p* = 0.001). The mean required time for performing the new method was 64 ± 10 s. An absence of the expected diffused dynamic view among ultrasound images obtained from patients with pneumothorax was also observed. We designated this phenomenon “Gestalt Lung Recession.”

**Conclusions:**

“Anterior convergent” chest US probing represents a brief but efficient model that provides clinicians a safe and accurate exam and adequate resuscitation during critical minutes of the primary survey without interrupting other medical staff activities taking place around the trauma patient. The use of the new concept of “Gestalt Lung Recession” instead of the absence of “lung sliding” might improve the specificity of US in detecting pneumothorax.

## Background

### Introduction

Despite the continued development of imaging technologies, pneumothorax (PTX) still represents a major concern among those who care for patients in emergency trauma settings or critical care systems. This condition can easily deteriorate into a life-threatening ailment if it is not diagnosed at the beginning of its course. The prevalence of PTX is greater than 20 % among severe trauma patients and up to 50 % among severe chest trauma patients [1, 2].

Applying a chest tube is a conventionally accepted therapeutic and diagnostic approach in patients whose clinical manifestations confirm a large or likely complicated PTX. Symptoms that indicate the diagnosis include shortness of breath and chest pain, and patients might appear ill or cyanotic and exhibit tachypnea; the signs include hypotension, decreased unilateral respiratory sounds and hyperresonance over the involved side, asymmetric chest expansion and subcutaneous emphysema or tachycardia. Nonetheless, a patient with a large PTX can appear misleadingly to be healthy [3].

Chest radiography has become the most popular diagnostic alternative in trauma settings. Most trauma guidelines, including ATLS (Advanced Trauma Life Support), recommend chest radiography in combination with anteroposterior pelvic radiography as adjuncts to the primary survey of trauma patients. However, several studies have noted the increased diagnostic value of ultrasonography compared to chest radiography for PTX [4–12].

Similar to our current model, in an unpublished online observation of American College of Emergency Physicians (ACEP) from December 2008, Arun Nagdev MM, explained the potential of ultrasound for detecting anterior traumatic PTX.

### Importance

Chest ultrasonography is safe, fast and noninvasive. Emergency physicians can perform the procedure before or in combination with the Focused Abdominal Sonography for Trauma (FAST) examination [7, 13, 14]. The combination is known and widely used today as EFAST (Extended Focused Abdominal Sonography for Trauma).

However, because of the relatively long performance time, neither EFAST nor its thoracic component is recommended or practiced during the primary survey. In addition, to avoid overlooking occult PTXs, many physicians perform a precipitate primary survey and resuscitation to enable early chest imaging incorporated with Advanced Trauma Life Support as adjuncts to the primary survey and resuscitation.

Developing a chest US protocol that is appropriate for the primary survey could ensure the efficient use of time for an accurate primary survey rather than introducing unnecessary interruptions in the breathing reassessment (B) or performing a hurried resuscitation with even less accurate imaging chest radiography (CXR). Moreover, an ideal protocol would not force other medical staff to interrupt their duties to protect themselves from radiation exposure.

### Goals of this investigation

Despite the advantages of chest US, few experiments have ever attempted or presented a chest US protocol appropriate for emergent moments in the primary survey. This time-directed performance study had two goals. First, we tried to develop a rapid chest US method, and second, we evaluated its performance accuracy and its time efficiency using an analytical approach.

## Methods

### Theoretical model of the problem

**Fig. 1:**
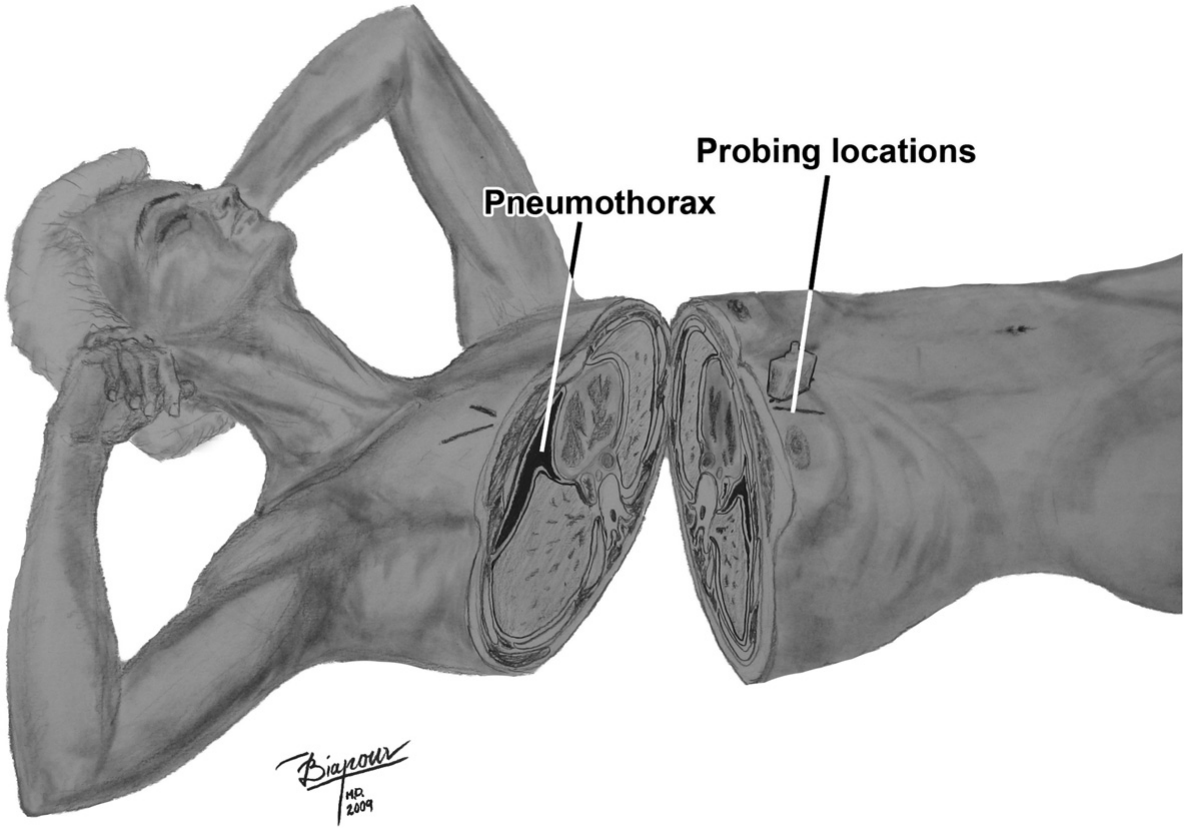
“Anterior convergent” chest ultrasonography for pneumothorax. In practice, the anatomical locations for probing provide sufficient views to detect even anterior apical or anterior basal entrapped air

**Fig. 2:**
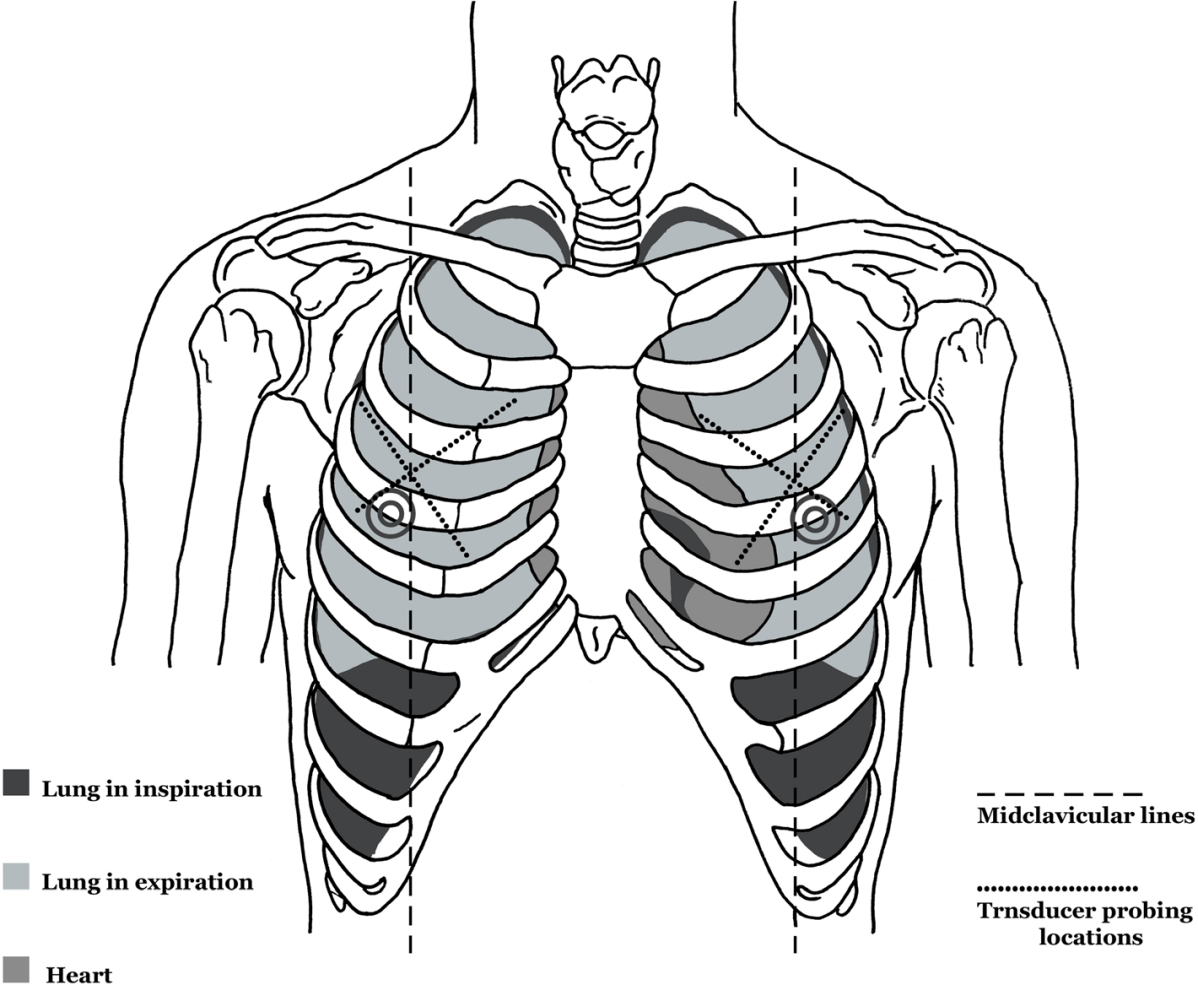
Anatomic guide to “anterior convergent” chest ultrasonography. The operator locates the transducer on the anterior chest wall over four convergent lines meeting near the intersection point of the midclavicular line and the third intercostal space

The physics concept that air accumulates at the most superior level in a closed space prompted the establishment of our relatively new and original but rapid method of transducer positioning. We used this principle in association with knowledge of the anatomical alignment of the ribs. Figure 1 outlines the concept of how the transducer positioning approach that we call “Anterior Convergent” enables the operator to detect even small pockets (<20 %) of likely entrapped air under the anterior wall of each hemi-thorax. In fact, the same reasons for selecting a needle decompression location provide the basis for the placement of the “Anterior Convergent” transducer [15]. Briefly, “Anterior convergent” is the transducer positioning protocol that obliges the operator to respectively locate the transducer on the anterior chest wall, over four convergent lines meeting near the intersection point of the midclavicular line and the third intercostal space Fig. 2.

### Study design, ethics and consents

This diagnostic trial was an experimental prospective study. The ethics committee of Tabriz University of Medical Sciences approved the study protocol without mandating any informed consent because the trial did not interfere with conventional diagnostic or therapeutic processes or with the patients’ autonomy in the emergency setting. No report of individual patient data was intended as well.

### Setting

The study was performed over 12 months from November 2008 to November 2009 in the resuscitation room of the emergency department. This crowded department has an average of 65,000 presentations per year, including both medical and trauma patients.

### Selection of participants

The study initially involved adult moderate or severe multiple trauma patients who had already been triaged and labeled as level 1, 2 or 3 by the Emergency Severity Index (ESI) Triage Algorithm Version 4. According to the ESI-4, patients who require an immediate life-saving intervention are labeled as level 1. The system labels patients as level 2 based on the following criteria: participation in high-risk situations, confusion, lethargy, disorientation, severe pain or distress or vital signs in the danger zone. Level 3, which accounts for the majority of moderate injuries, is applied to other patients than level 1 or 2. In this level, patients need more than one resource in their medical approach and do not have any vital sign in the danger zone.

**Fig. 3:**
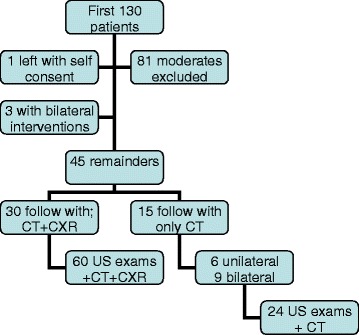
Case selection. The diagram depicts the case selection process and the number of patients involved in each preferred diagnostic imaging

As discussed below, we included hemi-thoraces in the study on a case-by-case basis. Ultrasound (US) results of either side were recorded independently from the contra-lateral side. Hemi-thoraces were excluded if a patient’s respiratory distress or clinical status mandated a PTX needle decompression or early chest tube placement before the computed tomography (CT) imaging. We had not yet proved the time efficiency of this first time trial. Thus, we could not ethically use this suggested adjunct to decide on such early interventions prior to the conventional clinical assessments. Hemi-thoraces in patients with severe chest wall pain who could not tolerate the placement of the transducer on their chest were also excluded. As presented in Fig. 3, all eligible hemi-thoraces were extracted from 130 patients who were sent to the trauma resuscitation room for primary exams.

In addition to one patient who left the ED against medical advice after the primary survey, the clinical status of three other patients required bilateral decompression of their hemothorax and/or PTX before the end of the primary survey.

A total of 81 patients who were primarily examined using US were considered to be moderate multiple trauma patients. Although they were excluded from the study, we believe that a comparison of their US results with their CXRs contributed to the optimization of the US operator’s level of expertise.

All 45 patients with severe chest injuries underwent ultrasonography followed by CXR and CT scanning. Because of the direct rapid disposition of 15 patients with substantial chest injuries from the ED radiology room to the surgery service, we did not have access to the radiography results of those patients. Nevertheless, a routine transfer of CT plots through the ED enabled the physicians in charge to report the results of the chest CT scans in these patients. Of these 15 patients, six underwent placement of a unilateral chest tube before CT imaging, due to their respiratory distress. Thus, hemi-thoraces with inserted chest tubes were excluded, and the contralateral hemi-thorax results were independently incorporated into the analysis.

To estimate the required procedure time, we used a pilot time study based on the time records from the previous 15 patients. Limiting the pilot sampling to the last 15 cases favored a more reliable time estimate when the operator had reached a constant and sufficient level of expertise based on his past chest ultrasonographies.

We determined how to collect the data before performing our index test US and the reference test (CT scan).

### Assignments

To avoid any inter-reviewer error, a single operator (corresponding author, emergency medicine resident PGY2-3) performed all the chest ultrasonographies. According to current standards, this operator had general emergency US competency derived from residency-based experience. Requirements to satisfy such standards include the performance of at least 150 US examinations in “critical” scenarios, rotation experience and supervised quality assurance with timely feedback during training [16]. After performing ultrasonographies on eight hemi-thoraces under the supervision of an attending physician, the operator then performed them independently. CTs of these training cases, with either normal or abnormal findings, were not included in the study, nor were their US results.

Based on the previously explained advantages of ultrasounds, all 130 patients who were sent to the resuscitation room underwent the anterior convergent chest US exam. The operator was required to perform the chest ultrasound exams as an adjunct to step B in the primary survey (Breathing; Ventilation and Oxygenation).

Chest CT scanning is generally considered to be the gold standard, particularly when the clinical status does not mandate an early chest tube insertion [17, 18].

Because exposing all patients to CT radiation is not ethically or medically accepted, the gold-standard application was limited to patients with severe multiple trauma who were routinely included in our hospital's whole body scan protocol. This protocol included spiral CT scans of the head, neck, thorax, abdomen and pelvis along with scanning of the skull, vertebral and pelvic bone windows for all multiple trauma patients with substantial injury mechanisms. Abdominopelvic IV contrast images were obtained for substantial injury mechanisms, including falls from > 20 ft; automobile crash with extrusion > 12 in. on occupant site or > 18 in. on other sites; ejection from the automobile; death in same passenger compartment; auto-pedestrian /bicyclist thrown, run over or with substantial impact; and motorcycle accident > 20 mph. This selection strategy allowed us to assess the results of each hemi-thorax, independent of the contra-lateral hemi-thorax status. For example, of six patients with unilateral chest tubes inserted during the primary survey, only six hemi-thorax CT scan results were included in the analysis. Finally, patients underwent CT scans if they were sufficiently hemodynamically stable to be transferred out of the emergency department for imaging.

Consequently, moderate chest injuries were solely imaged using CXR after the primary survey during the phase known as Adjuncts to the Primary Survey and Resuscitation.

Chest US, radiography and CTs were all performed with the patient in a supine position. The US operator reported his results before learning of the radiography and CT results. CXRs and CTs were reported by physicians who were responsible for performing the secondary surveys. All these physicians were PGY2 emergency medicine residents, and they were all blinded to the chest US findings. The overall independent results of the imaging interpretations were recorded as positive or negative for PTX based on primary check lists. CT reports were noted in the checklist if the scan results were recorded within a one-hour interval after the US exam.

The recording times of the procedures were determined by the operator who used the US machine screen timer. The time between the first contact of the transducer over the first hemi-thorax until the transducer was removed from its last location on the second hemi-thorax was defined as the procedure time.

### Methods of measurement

All present PTX ultrasound findings were based on features reflecting images from superficial pleural components. Therefore, to achieve our goal of a rapid exam, we modified the conventional US exam to include only B-mode images obtained by a 9-MHz linear transducer. The transducer was positioned over four locations above the anterior wall of each hemi-thorax. Each location was capable of providing the operator with a lung view through the window of at least one intercostal space. These four linear positions generally created a radiation appearance around a point where the midclavicular line intersected with the third intercostal space. The shortest depth to the pleura was expected at these locations (Fig. 1).

**Fig. 4:**
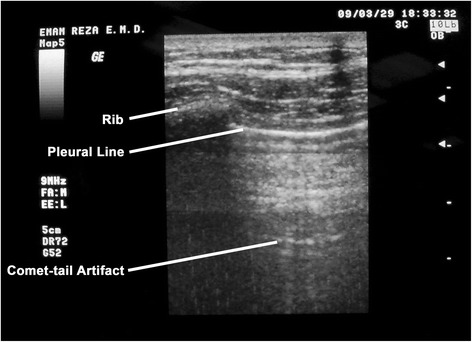
Ultrasound pattern of the normal lung in B-mode with a linear transducer. The pleural line is depicted as the hyper echoic line limited between two sequential ribs. The backward and forward movement of pleural layers against each other occurs on the same level as the pleural results in lung sliding. The comet-tail artifact is a reverberation artifact arising from the pleural line and appears as a hyper echoic line parallel to the pleural line and at the depth of the parenchyma

Our chest US operator utilized the currently available US indicators that suggest a PTX, namely, the “pleural line”, which is defined as a horizontal hyper-echoic line between the ribs [4, 19]; the “sliding sign” (or gliding sign), which is visualized as a back-and-forth movement during the respiratory cycle and is caused by the visceral and parietal layers moving against each other [20–23]; and the “comet-tail artifact,” which is known as a hyper-echoic reverberation artifact that reflects the pleural line and is thought to rule out PTX [23–26] (Fig. 4).

### Outcome measures

The primary focus of the study was to determine the sensitivity of our newly established method for chest US probing. Other diagnostic characteristics will be described below according to the following formulas [27, 28]:

$$ \mathrm{Sensitivity} = \mathrm{True}\ \mathrm{Positive}\ \mathrm{Rate} = \frac{TP}{TP+FN} $$

$$ \mathrm{Specificity} = \mathrm{True}\ \mathrm{Negative}\ \mathrm{Rate} = \frac{TN}{TN+FP} $$

$$ \mathrm{Positive}\ \mathrm{Predictive}\ \mathrm{Value} = \mathrm{probability}\ \mathrm{that}\ \mathrm{the}\ \mathrm{disease}\ \mathrm{is}\ \mathrm{present}\ \mathrm{when}\ \mathrm{the}\ \mathrm{test}\ \mathrm{is}\ \mathrm{positive} = \frac{TP}{TP+FP} $$

$$ \mathrm{Negative}\ \mathrm{Predictive}\ \mathrm{Value} = \mathrm{probability}\ \mathrm{that}\ \mathrm{the}\ \mathrm{disease}\ \mathrm{is}\ \mathrm{not}\ \mathrm{present}\ \mathrm{when}\ \mathrm{the}\ \mathrm{test}\ \mathrm{is}\ \mathrm{negative} = \frac{TN}{TN+FN} $$

Moreover, the evaluation of all previously explained ultrasound diagnostic features, including the sliding sign, during one respiratory cycle was expected. A complete respiratory cycle lasts approximately five to ten seconds in a normal adult (with six to twelve respirations per minute). Thus, with regard to the use of eight locations, the entire US procedure on a single patient theoretically could be completed within 40 to 80 s. The mean required time for a single US application was considered our secondary outcome measure.

### Data collection and processing

The chest US, CXR and chest CT scan results from 84 hemi-thoraces were recorded and saved in SPSS 15.0 /_Win_. The PTX results included in the obtained cross-tabulations (Tables 1 and 2) were the initially processed results, and the chest ultrasound and chest radiography with chest CT scan findings were compared. The results from each hemi-thorax were independently incorporated into the calculations and estimations. To avoid correlated data between hemi-thoraces, the US diagnostic values were determined independently of the PTX prevalence of any particular chest trauma level of severity. P-values less than 0.05 were considered to be statistically significant for all results.

**Table 1:** Cross-tabulation of the ultrasound results by CT scan for pneumothorax

		Results for pneumothorax		Total
		Negative CT scans	Positive CT scans	
Results for pneumothorax	Negative ultrasounds	61	4	65
	Positive ultrasounds	5	14	19
Total		66	18	84

**Table 2:** Cross-tabulation of chest radiography results by CT scan for pneumothorax

		Result for pneumothorax		Total
		Negative CT scans	Negative CT scans	
Results for pneumothorax	Negative chest radiographies	48	7	55
	Positive chest radiographies	1	4	5
Total		49	11	60

The procedure times were saved in a Word file for subsequent calculations using relevant software.

### Primary data analysis

We used the findings of Zhang et al. [4] to estimate the sample size, which was computed using the Pearson chi square test for two proportions and with the elements fixed as Alpha = 0.05 and nominal power = 80 %.

Because our study evaluated the screening capabilities of a test, the sample size with a total number of 76 cases was calculated using the SAS system for a sensitivity of 0.86 (for proportion 1; US) and 0.27 (for proportion 2; CXR) and an actual power of 0.81, which were all extracted from the study by Mao Zhang et al. An ideal screening test is closely dependent on its sensitivity, as will be addressed in the “Discussion”. However, to increase the validity of the study and further exclude exams throughout the trial, we continued collecting samples until the overall number of 84 exams was reached.

## Results and discussion

### Main results

According to the contents of Table 1, of 84 results obtained using chest US exams for detecting PTX, 14 were true positives (TPs), 61 were true negatives (TNs), five were false positives (FPs), and four were false negatives (FNs).

Similarly, of 60 CXR results as presented in Table 2, four TP, 48 TN, one FP and seven FN results were extracted.

A mean required time of 64 s for performing the US exam on each patient was calculated with a standard error of the mean (SEM) of 10 s.

As explained above, the “sliding sign” represents a linear back-and-forth movement that disappears in the presence of PTX. Nonetheless, in practice, the immobilized PTX US views appeared to exhibit more than a simple absence of linear movement. Instead, they appeared to present a diffuse static background. Consequently, as the inductive part of our research, we called this phenomenon the “Gestalt Lung Recession.”

### Sensitivity analysis

Diagnostic values were calculated when the above-obtained quantities were entered into MedCalc software (Ver. 10. 4.; Frank Schoonjans). The same software produced predictive values based on the use of Fisher’s exact test (Table 3). The kappa agreement coefficient indicated a very acceptable inter-reviewer validity of > 0.8 for the reports (*p* < 0.001) [29].

**Table 3:** Diagnostic characteristics of index tests for pneumothorax

	Chest ultrasound		Chest radiography		Comparison
Parameter	Value	95 % CI	Value	95 % CI	*P*-Value
Sensitivity	78	52–93	36.4	11–69	<0.001
Specificity	92	83–97	98	90–100	0.1
LR+	10	4.3–25	18	2–144	0.007
LR-	0.25	0.1–0.6	0.65	0.4–1	<0.001
PPV	74	49–91	80	28–99	0.4
NPV	94	85–98	87	75–95	0.14

LR+: positive likelihood ratio; LR-: negative likelihood ratio; CI: confidence interval. All values are expressed as percentages, except for LR+ and LR-

### Limitations

Assuming that a PTX diagnosis is not a core emergency US application [17], our inability to comply with the completion of the US training over 25–50 cases was a limitation. Fortunately, as the study progressed, we found previous research that suggested that one didactic one-hour session was sufficient for improving PTX recognition skills [30]. A spontaneous PTX case during our short supervised training schedule was a valuable opportunity for our operator to evaluate the presentation of PTX, which occurs infrequently. We offer US training using artificial PTXs to practitioners who intend to reproduce our model, although we do not have the appropriate setting for the training [31, 32]. The “emergency ultrasound guideline” we used and offered during our study is the minimal requirement and basic standard for performing an US examination. However, because an US examination is an experience-dependent skill, an operator with more experience is expected to exhibit better practice.

Estimating the PTX volume requires determining the borders at which the sonographic PTX features disappear [33]. To determine this point at which a normal lung pattern replaces the PTX pattern, known as the “lung point,” each examination needs to be extended to the lateral regions of the chest wall [19]. Without extension to the lateral chest walls, an “Anterior Convergent” chest US is unable to provide an estimation of the PTX volume. However, because the volume estimation could be time consuming, it contradicted the main purposes of the study.

US diagnostic findings also have inherent limitations. The comet-tail artifact is usually known as a pathologic finding [34], and its absence might be expected in completely normal lung parenchyma. “Lung sliding” might disappear when an adhesion of parietal and visceral pleura exist [14] as well as in conditions such as bullous emphysema and advanced chronic obstructive pulmonary disease [5]. Such limitations might be able to explain how five false-positive results occurred among our 84 exams. Conversely, chest wall contractions should be distinct from “lung sliding.” Finally, subcutaneous emphysema can cause a poor view, and unusual locations of entrapped air might theoretically be masked from being detected in our model. All such mechanisms could have contributed to our four false-negative results.

Moreover, it should be acknowledged that a larger sample size could have increased the power of our study.

### Discussion

The CXR specificity of 98 % and the positive predictive value of 80 % were similar to the findings of our chest US exam, which were 93 % and 74 %, respectively, and the differences were not significant (*p* = 0.1). The “Anterior Convergent” chest US demonstrated a sensitivity that was significantly superior to that of CXR (78 % versus 36 %, respectively, with *p* < 0.001). Although they are not values, the diagnostic characteristics of “Anterior Convergent” chest probing exhibited the same accuracy pattern based on the available studies that investigated the conventional models of detecting PTX using US.

The clinically valuable high NPV of 93 % indicates that a negative US result can strongly rule out PTX. Of course, the NPV of CXR was acceptable and did not indicate a significant difference from the US NPV (*p* = 0.14). However, the US diagnostic values are related to the clinical importance of both “sensitivity” and “specificity.” Tests with high specificity are required when the risky consequences of a false-positive diagnosis are a primary concern. Chemotherapy following a falsely diagnosed malignancy is an example. In fact, highly specific tests are optimal for ruling in diseases, and we believe that CXR is still a valuable test in such instances. However, a test with a high sensitivity is obviously required when a patient is exposed to a high risk by a potential false-negative diagnosis. Patients with occult PTX who require either bag mask ventilation during the primary survey or mechanical ventilation just after their primary survey account for such conditions because they can plainly develop a life-threatening tension PTX under any positive pressure ventilation. In fact, highly sensitive tests are ideal screening tests for ruling out diseases [35, 36].

As a result, until diagnostic alternatives with higher sensitivity are developed, chest US remains the superior bedside screening test for detecting PTX in supine trauma patients.

We believe that chest US using the “Anterior Convergent” probing method constitutes a feasible model for clinical practice, particularly during critical moments of the primary survey. The pilot result of 64 s for the mean required time was considerably lower than the estimations of two to four minutes to perform formal chest US [5]. We particularly recommend optimization of the related sensitivity. Serial US exams during the secondary survey using the “Anterior Convergent” model appear to be a viable option [37]. Adding a single thoracic US using conventional whole-chest probing and integrating it into FAST of the secondary survey might be another strategy.

The minimal probing and transducer positioning were required in practice as we expected from the “Anterior Convergent” model. Nevertheless, we intend to explain some other aspects of this experience to assist clinicians who intend to use our model in their practice.

A pre-set depth of five to six centimeters on the US machine provided a convenient view in most patients. Performing an accurate US exam generally appeared to be easier among males and small patients than on females or obese patients. The breast tissue was implicated in the poor view in the female patients. Different layers of the chest wall could easily resemble the “pleural line,” particularly for a non-expert operator. Our overall concept of “lung sliding” was the indicator with the lowest specificity but the highest sensitivity [38]. The presence of this sign contributed to the quickest ability to rule out the presence of PTX, whereas the absence of this sign is unreliable for ruling in the diagnosis.

Furthermore, we investigated “lung sliding” beyond the traditional definition of the sole pleural movement. Shadows and artifacts associated with the pleura induce a back-and-forth movement of the entire image background. In the presence of PTX, the absence of such a diffuse dynamic appearance creates a static view that we named the “Gestalt Lung Recession.” The “Gestalt Lung Recession” appeared to be more specific for ruling out PTX than the absence of “lung sliding.”

The performance time appeared to be shorter among patients with tachypnea and relatively prolonged in patients with bradypnea or a poor view, including those with subcutaneous emphysema.

Despite the growing body of evidence confirming the advantages of chest US in the diagnosis of PTX, a general hesitation is still apparent in global guidelines. The American College of Emergency Physicians (ACEP) has recommended the use of ultrasound for the detection of PTX but did not provide the details of such methods [16], and the American College of Surgeons (ACS) has not included thoracic US in the 9^th^ edition of ATLS [39]. Therefore, future studies should review the literature for all unidentified aspects of chest US, either independently or in conjunction with meta-analyses. Such reviews should include the available models of chest US and comparisons of the models and the diagnostic presentation of PTX for all available model types. Such a comparison should include all aspects of accuracy and time effectiveness as well as the performance feasibility of each method or the recognition indicators for different conditions. If such studies are capable of explaining the above-mentioned uncharted issues, they will break through the current trauma guidelines and ATLS.

Our study would have provided much more raw data for future analysis if it had been primarily designed to repeat the “Anterior Convergent” protocol during the secondary survey. If that strategy had been employed, the sensitivity of both single-practiced and duel-practiced “Anterior Convergent” chest probing could have been compared. Any higher level of sensitivity for the dual-practiced model would have emphasized the “Anterior Convergent” model as the ideal method for detecting PTX during the primary survey.

## Conclusion

Ultimately, we believe that “Anterior Convergent” chest probing is a practical method for detecting PTX. This approach is a more appropriate screening test than both supine conventional CXR and lung auscultation. We particularly recommend using this brief but efficient technique during the emergent minutes of the primary survey in trauma patients who present to the emergency department, and we currently use this application in our center.

It is reasonable to predict that clinicians will one day consider US machines with the same familiarity they currently feel for their stethoscopes, although US machines are more sophisticated.

## Abbreviations

ACEP: American College of Emergency Physicians
ACS: American College of Surgeons
ATLS: Advanced Trauma Life Support
CT: computed tomography
CXR: chest radiography
ED: Emergency Department
EFAST: Extended Focused Abdominal Sonography for Trauma
FAST: Focused Abdominal Sonography for Trauma
FN: false negative
FP: false positive
LR: Likelihood ratio
NPV: negative predictive value
PPV: positive predictive value
PTX: pneumothorax:
SEM: standard error of the mean
TN: true negative
TP: true positive
US: ultrasound
